# Distribution of Cytomegalovirus Genotypes among Neonates Born to Infected Mothers in Islamabad, Pakistan

**DOI:** 10.1371/journal.pone.0156049

**Published:** 2016-07-01

**Authors:** Ghulam Mujtaba, Adnan Khurshid, Salmaan Sharif, Muhammad Masroor Alam, Uzma Bashir Aamir, Shahzad Shaukat, Mehar Angez, Muhammad Suleman Rana, Massab Umair, Aamer Ali Shah, Syed Sohail Zahoor Zaidi

**Affiliations:** 1 Department of Microbiology, Quaid-i-Azam University, Islamabad, Pakistan; 2 Department of Virology, National Institute of Health, Islamabad, Pakistan; CEA, FRANCE

## Abstract

**Background:**

Congenital cytomegalovirus (cCMV) infection contributes to considerable long-term sequelae in neonates and children all over the world. The association between viral genotypes and severity of clinical cytomegalovirus (CMV) infection is yet to be defined. The objective of this study was to find the impact of active CMV infection during pregnancy and the clinical significance of genotypes in neonates with congenital cytomegalovirus infections in Pakistan.

**Methods:**

A total of 409 blood samples from pregnant women seeking health care services at the two antenatal hospitals of Islamabad during January to December 2012 were tested by ELISA and nested-PCR. Pregnant women with active infection (detected as IgM positive, PCR positive or positive on both assays) were followed until delivery, to detect the outcome of overt cCMV infection in neonates. Genetic characterization of CMV strains was performed by sequence analysis of envelope glycoproteins: gB, gN and gH to detect the contributing CMV genotypes.

**Results:**

The seroprevalence of anti-CMV IgG and IgM was 97.5% (399 out of 409) and 12.7% (52 out of 409), respectively, while 20% (82/409) pregnant women were found positive for CMV DNA by PCR. Logistic regression analysis showed a significant association of active infection with parity [OR = 2.56, 95% CI = 1.82–2.62, *p* = 0.04], febrile illness [OR = 1.84, 95% CI = 1.76–3.65, *p* = 0.01] and jaundice [OR = 22.5, 95% CI = 4.53–85.02, *p* = 0.002]. We were able to isolate virus in 41 out of 70 neonates; 36.6% (15 out of 41) of them were symptomatic at birth while 63.4% (26 out of 41) were asymptomatic. The most prominent clinical feature observed in symptomatic neonates was hepatosplenomegaly (26.6%; 4 out of 15). All three genotypes gB, gN and gH were found with the highest frequency of gB1 genotype, found in 75% infants with hepatic damage. Phylogenetic analysis of Pakistani strains showed 96%-100% homology to their prototype strains.

**Conclusions:**

Active CMV infection during pregnancy is a major cause of congenital CMV infection with comparable distribution of all three genotypes: gB, gN and gH in symptomatic and asymptomatic neonates. Our findings emphasize to conduct a comprehensive large scale survey and introduction of country wide routine screening at maternity clinics for early diagnosis of CMV to reduce its associated devastating outcomes.

## Introduction

Human cytomegalovirus (HCMV) belongs to the *beta herpesvirinae* family, and humans are its only natural hosts. HCMV, also called human herpesvirus-5 (HHV-5), is one of the 8 human herpesviruses. HCMV is a common cause of congenital cytomegalovirus (cCMV) infections in developed as well as developing countries. Cytomegalovirus (CMV) infection may be acquired prenatally (congenital) through transplacental acquisition of either a primary or a recurrent maternal infection or during the perinatal/postnatal phase—at the time of birth or immediately after, due to exposure to infected cervical secretions, breast milk, or blood products [1]. The severity of congenital infection depends on maternal primary infection or re-activation, with primary infection more likely to result in severe sequelae. In developing countries, mostly congenitally infected neonates born to women with recurrent infections are clinically asymptomatic [2].

HCMV is a large, genetically diverse virus with over 200 open reading frames [3]. Genotyping of HCMV is principally based on variation in surface glycoprotein sequences, which show frequent genetic polymorphism. The HCMV genome encodes numerous glycoproteins; gB, gH and gN are the most abundant and have been studied extensively. Glycoprotein B (gpUL55) is a polymorphic glycoprotein and is a component of envelope complex gB-I with four genotypes (gB1-gB4) [4, 5]. The gH glycoprotein, an 86 kDa protein, is encoded by the UL75 gene and has two major variants—gH1 and gH2, based on the variability in the 37 amino acid N-terminal domain [6]. Another HCMV surface glycoprotein, gpUL73 (gN), encoded by UL73 has four genomic variants termed gN-1, gN-2, gN-3 and gN-4, with gN-3 sub-divided into gN-3a and gN-3b while the gN-4 genotype has three subgroups (gN-4a, gN-4b and gN-4c), respectively[7].

Several studies have been carried out to verify an association between genotype and disease manifestation, but heterogeneous findings from the various reports failed to define clear linkages [8, 9]. Approximately 10% of infants with cCMV exhibit clinical symptoms at birth, including intrauterine growth retardation (IUGR), jaundice, hepatosplenomegaly, retinitis, purpura, seizures and thrombocytopenia [10, 11]. Among the remaining 90% of infants that are asymptomatic at birth, 8% to 15% later develop complications, mainly neuro-developmental defects and deafness [12]. Thus, there is a need for early diagnosis, close monitoring, and timely therapeutic interventions to avoid the development of serious consequences in these asymptomatic children. Furthermore, HCMV infections can be a predisposing factor for bacterial and fungal infections due to reduced immune response and may have a role in graft rejection [13].

The prevalence of congenital CMV infection is usually higher in developing countries with higher maternal seroprevalence and varies within and between countries, with some reports as high as 6–14% [14]. There is currently no published data regarding cCMV infection in Pakistan. The aim of the present study was to identify cCMV infection, determine prevalent genotypes and assess possible relationships between genotypes and clinical features of the neonates born to actively infected mothers.

## Materials and Methods

### Study design

Pregnant women seeking health services at two major antenatal public sector hospitals of Islamabad, Federal Government Services Hospital and Pakistan Institute of Medical Sciences, during January to December 2012 were enrolled in this study. Furthermore, pregnant women with active infections were followed up to identify cCMV infection and genetic variability among circulating genotypes in neonates. Inclusion criteria of this study were: (1) Maternal active infection documented by either serum CMV-IgM antibody and/or PCR positive results; (2) Congenital CMV infection demonstrated by virus isolation within 3 weeks of age for neonates.

Demographic and clinical data were collected from the medical reports of newborns and from parents with their permission. The purpose of study was explained, and written consent was obtained from all participants. For minors and children, an informed written consent from the parents, caretakers, and/or guardians was obtained after explaining the study details and outcomes. The laboratory diagnostic and sequencing work was carried out at the Department of Virology, National Institute of Health (NIH), Islamabad. The study design and concept was approved by the ethical committee of respective Hospitals and of the Internal Review Board of the National Institute of Health (NIH), Pakistan.

### Sample Collection and Processing

At the time of enrollment, blood samples were collected from all enrolled pregnant women (n = 409). During the follow-up visits, five cc of blood sample was collected from mothers. From neonates, two types of samples, urine and saliva, were collected within the fifteen days of their birth. Saliva was collected from all enrolled infants (n = 70) and urine was collected from 18 neonates within fifteen days of their birth.

Urine samples were collected in sterile urine bags attached to the perineum and transported on ice to the laboratory. Saliva samples were collected by swabbing the inside of neonates mouth using sterile polyester fiber tipped applicator and transported on ice to the laboratory. The saliva samples, when received, were incubated at 55°C for 1 hour with lysis buffer containing proteinase K, followed by denaturation of the enzyme at 95°C for 10 minutes.

### Serology

To determine the immune status (IgG and IgM) of each participant and to calculate the anti-HCMV IgG avidity index, a commercial diagnostic ELISA kit (HUMAN Gesellschaft für Biochemica und Diagnostica mbH, Germany) was used. Blood samples were processed according to the manufacturer’s protocol, and results were interpreted accordingly.

### Detection of HCMV by PCR

DNA was extracted from plasma, saliva and urine using a Nucleospin® Blood kit (MACHEREY-NAGEL GmbH & Co. KG, Germany) according to the manufacturer's protocol and stored at -20°C until further use. Nested PCR was carried out for screening in a 50 μl volume of 10X PCR buffer, 2.5 mM MgCl_2_, 200μM of each dNTPs, 5 units of *Taq* DNA polymerase and 10pmol of 2 primer sets specific for the 1st and 2nd round of amplification as described previously [15]. The cycling conditions for both rounds were as follows: 35 cycles at 94°C for 30 seconds, 58°C for 40 seconds and 72°C for 50 seconds and terminal extension at 72°C for 3 minutes. The amplified products were electrophoresed on a 2% agarose gel containing ethidium bromide and visualized under UV trans-illuminator.

### Characterization of CMV gB, gN and gH genotypes

The samples positive for CMV were further subjected to nested PCR to amplify the UL55 (gB), UL73 (gN) and UL75 (gH) genes for genotyping [6, 16, 17]. All samples were tested for the detection of all three genotypes (gB, gH or gN). The primers and thermal conditions used for amplification are given in Table 1.

**Table 1:** Oligonucleotide primers and thermal conditions used for PCR and sequencing of cytomegalovirus.

Target gene-Primer name	Sequence (5'-3')	Initial denaturation	Annealing temperature	Elongation time	Reference
UL55(gB)-Outer Forward	TCCGAAGCCGAAGACTCGTA	94°C/5min	58°C /1 min	72°C/5 min	[16]
UL55(gB)-Outer Reverse	CATTCCTCAGTGCGGTGGTT				
UL55(gB)-inner Forward*	TTTGGAGAAAACGCCGAC	94°C/2 min	60°C /1 min	72°C/5 min	
UL55(gB)-inner Reverse*	CGCGCGGCAATCGGTTTGTTGTA				
UL73(gN)-outer Forward	GACAGTACCAGTTGAGAGTCG	96°C/5 min	55°C /1min	72°C/2 min	[17]
UL73 (gN)-outer Reverse	GGAYTATCTAGACTCGCTGC				
UL73 (gN)-inner forward*	TGGTGTGATGGAGTGGAAC	94°C/2 min	60°C/2min	72°C/1 min	
UL73 (gN)-inner reverse*	TAGCCTTTGGTGGTGGTTGC				
UL75(gH)-outer Forward	CCTTCTCTCGGGTGTAAGC	96°C/5 min	55°C /1min	72°C/2min	[6]
UL75(gH) outer Reverse	GTAGGTGTTAAGTCTCTG				
UL75(gH) inner Forward*	CCACCTGGATCACGCCGCTG	94°C/2 min	60°C/2min	72°C/1 min	
UL75(gH) inner Reverse*	TGGTGTTTTCACGCAGGAA				

Y = C+T (Based on the CMV strain AD169 sequence)

* Primers used for sequencing

### Phylogenetic analysis

The amplicons of UL55, UL73 and UL75 were purified using a QIAquick PCR Purification kit (Qiagen) and sequenced in both directions using a BigDye Terminator v3.1 Cycle Sequencing kit (Perkin Elmer-Applied Biosystems, Inc., Foster City, CA, USA). The sequences were analyzed on an ABI Prism Genetic Analyzer (3130xl, Applied Bio systems) and edited by Sequencher v.4.1 (Gene Codes Inc., Ann Arbor, MI, USA). Phylogenetic analysis of the UL55, UL73 and UL75 genes were performed using strains of different genotypes and of different geographical regions as retrieved from GenBank. Evolutionary tree and distances were generated by the neighbor-joining method using MEGA 5.0 [18].

### Statistical analysis

The analysis was carried out using SPSS v.18.0. The mean (± Standard Deviation) values were calculated for age, and frequencies and percentages were calculated for HCMV positive and negative subjects. Various parameters were compared between HCMV positive and negative cases. Student’s t test was used for comparison of continuous variables, and the Pearson chi-square test was used for comparison of dichotomous variables. Logistic regression analysis along with calculation of odds ratio (OR) and 95% confidence intervals (CI) were performed to determine the association between the characteristics of subjects and HCMV seropositivity or active infection. Level of statistical significance was p < 0.05 (two-sided).

## Results

A total of 409 pregnant women were enrolled in this study with over half (67.2%) of them residing in rural areas of Islamabad. The majority (96.82%) of pregnant women enrolled in this study were housewives while 3.18% were working women. The mean age (±SD) was 26.8 (±3.8) years with a range of 16–40 years.

### Socio-demographic, obstetrical and clinical features of pregnant women

One hundred and eighteen (28.8%) women had a past abortion and thirty-four (8.31%) reported a history of jaundice. At the time of enrollment, 45 (11%) women were in the first trimester, 160 (39.1%) were in their second trimester, while 204 (49.87%) women were in their third trimester. One hundred and forty-eight (36%) of the women were pregnant for the first time (primigravida) while two hundred and sixty-one (64%) were multiparous. Logistic regression analysis of the various factors showed that high parity (>3 deliveries) [OR = 2.56, 95% CI = 1.82–2.62, p = 0.04] had a significant association with active CMV infection in these women. However, other parameters such as maternal age, area of residence, education level, trimester of pregnancy, and history of previous miscarriages were found without a significant effect (Table 2).

**Table 2:** Logistic regression analysis of demographic and clinical features of mothers infected with active CMV infection.

Features	Odds ratio^a^	95% confidence interval	*P*-value
Age (years)			
16–30	0.45	0.89–3.32	0.18
31–40	0.04	0.97–2.80	0.15
More than 3 children	2.56	1.82–2.62	0.04
History of jaundice	22.5	4.53–85.02	0.002
Febrile illness	1.84	1.76–3.65	0.01
Gestational trimester	1.08	0.64–1.25	0.1
History of abortion	0.78	0.45–1.08	0.2
Education level	0.26	0.87–1.58	0.3
Residence	0.87	0.36–1.67	0.5
History of abortion	0.63	0.58–1.42	0.09
Hemoglobin	0.49	0.96–1.36	0.4

^a^ Adjusted by the features included in this table

OR = Odd Ratio

CI = Confidence Interval

### Detection of IgM, IgG and HCMV DNA

Among the 409 pregnant women enrolled in the study, 399 (97.55%) were positive for IgG antibodies while IgM antibodies were detected in 52 (12.71%) women. Using molecular testing, HCMV DNA was detected in only 82/409 (20%) of the women. Eighty two (n = 82; 20) women were found with active infection (either positive on IgM ELISA and/or PCR) as given in Table 3.

**Table 3:** Results of IgM, IgG and PCR testing for diagnosis of CMV infection in pregnant women.

	Serology Results				
PCR results	IgM+ IgG+ n(%)	IgM+ IgG- n(%)	IgM- IgG+ n(%)	IgM- IgG- n(%)	Total n(%)
PCR +ve	48 (58.5)	2 (2.4%)	32 (39%)	0	82 (20%)
PCR -ve	2 (0.6%)	0	315 (96.3%)	10 (3%)	327 (80%)
**Total**	50	2	347	10	409

**Fig 1:**
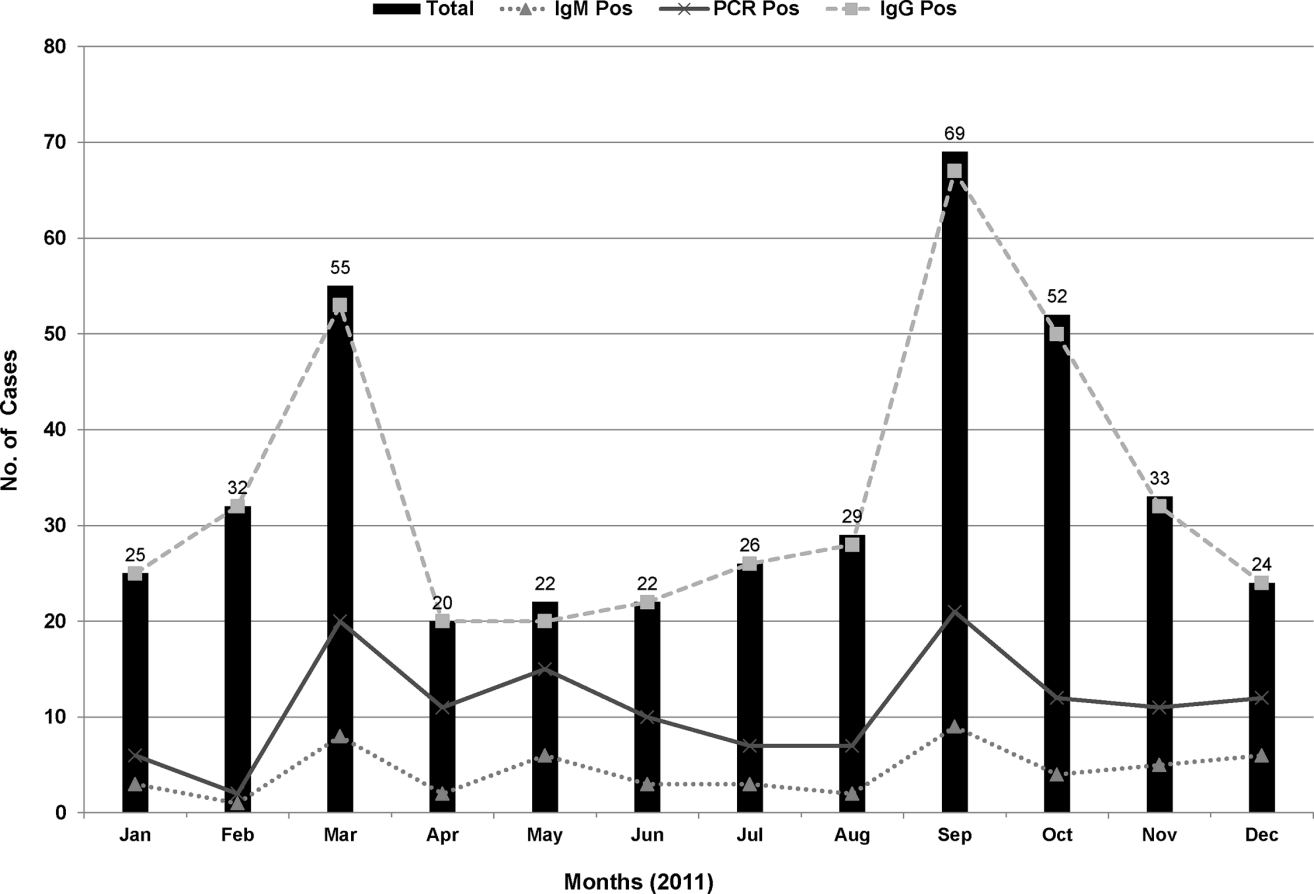
The distribution of HCMV positive cases in study population during 2012. The months are given on X-axis. The data labels on Y-axis indicate the number of total and positive cases across the year. The solid black line indicates total number of cases while the number of positive cases for each assay indicated with different bar-style.

Patients with active infection were defined as “those with either IgM Positive only, or PCR positive only or both IgM and PCR positive” as demarcated by CDC, USA (http://www.cdc.gov/cmv/clinical/lab-tests.html). For the clinical characteristics, (fever) [OR = 1.84, 95% CI = 1.76–3.65, p = *0*.*01*] and history of jaundice [OR = 22.5, 95% CI = 4.53–85.02, p = *0*.*002*] had significant associations with active CMV infection in pregnant women. Other clinical features such as lymphadenopathy, sore throat and flu showed no significant association with active CMV infection. No seasonal trend of CMV infection was observed; however, it was more frequent in March and September of the study year (Fig 1). The pregnant women who were seronegative (2.4%) were unprotected and fall within the group in which infection is likely to occur.

IgG avidity assay was performed on 52 IgM positive samples and found that six samples showed low HCMV IgG avidity (<50%), two samples fell in grey zone (54% index) and 42 samples presented with high avidity (>60%). These findings confirmed that six women showed primo-infection and 44 (84.6%) had secondary infection.

### Outcomes of congenital CMV infection

Eighty-two (n = 82) pregnant women showing active infections were followed up to study congenital infection in neonates. Out of these, five (n = 5) pregnancies were aborted or intrauterine fetal deaths occurred, while seven cases were missed as we were unable to follow up or refused to participate further. Seventy (n = 70) pregnancies were followed up successfully until deliveries for the congenital CMV infection study. Out of these cases, the virus was isolated from forty-one (n = 41) neonates within three weeks of age. Fifteen (n = 15) neonates were symptomatic while twenty-six (n = 26) were asymptomatic at birth. There were 6/10 (60%) symptomatic neonates born to mothers with first trimester infections compared with 9/60 (15%) born to mothers with infections in the second and third trimester, indicating significant association between the stage of mothers exposure and the clinical infections in newborns (Table 4). The proportion of clinically infected newborns whose mothers got infected during the first trimester was higher than those whose mothers were found CMV positive during second and third trimester thus indicating a strong association of the gestational age of mothers’s infection with the appearance of clinical disease in the newborns.

**Table 4:** Association of clinical infection in newborns compared to the mother’s gestational stage of CMV exposure.

Maternal CMV exposure	Diseased	Not Diseased	Total	Odd ratio	95% CI	P-value
**First trimester**	6	4	10	8.5	1.993–36.25	0.0048
**Second +Third trimester**	9	51	60			
**Total**	15	55	70			

When clinical manifestations of symptomatic newborns were compiled, it was found that hepatosplenomegaly (4/15; 26.6%) was the most common feature followed by neonatal jaundice (3/15; 20%) and premature delivery (Table 5). No significant differences were found between asymptomatic and symptomatic infants with respect to maternal clinical and demographic parameters. Among the clinical samples collected and tested for CMV infection in neonates, high frequency of CMV infections were detected through saliva samples compared to urine. Out of 18 urine samples, 5 (27.7%) were positive while 100% of saliva samples were positive for CMV.

**Table 5:** Clinical Abnormalities found in Newborns with Symptomatic Congenital CMV Infection.

Findings/Abnormality	Positive/Total Examined (%)
Bronchopneumonia	1/15 (6.6)
Congenital Cataracts	1/15 (6.6)
Developmental Delay	1/15 (6.6)
Hepatosplenomegaly	4/15 (26.6)
Hydrocephaly	1/15 (6.6)
Thrombocytopenia (Platelet count <100000/mm3)	2/15 (13.3)
Microcephaly	1/15 (6.6)
Neonatal Jaundice	2/15 (13.3)
Respiratory Distress	1/15 (6.6)
Petechiae	1/15 (6.6)
Petechiae with Jaundice	1/15 (6.6)

### Distribution of HCMV genotypes and phylogenetic analysis

A total of 41 PCR positive newborns were further analyzed for genotype determination. The three major genotypes were detected in 36 cases while PCR analysis for HCMV genotype was negative in five cases. Sequence data on representative strains of the various genogroups were retrieved from GenBank and included in the sequence alignment and phylogenetic analysis. All four gB genotypes (1–4), six gN sub-genotypes (1, 3a, 3b, 4a, 4b, 4c) out of 7 and both gH genotypes (1 and 2) were detected in the study population, with variable proportions as described in Table 6. Majority of the isolates were identified as the gN (15; 41.6%) genotype while 11 were identified as gH (30.5%) and 10 as gB (27.7%).

**Table 6:** Genotype distribution of CMV in neonates born with congenital infection.

Infants	gB1	gB2	gB3	gB4	gH1	gH2	gN1	gN-3a	gN-3b	gN-4a	gN -4b	gN-4c	Total
Bronchopneumonia	0	1	0	0	0	0	0	0	0	0	0	0	1
Congenital Cataracts	0	0	0	0	0	0	0	0	1	0	0	0	1
Developmental Delay	0	0	1	0	0	0	0	0	0	0	1	0	1
Hepatosplenomegaly	3	0	0	0	0	1	0	0	0	0	0	0	4
Hydrocephaly	0	1	0	0	0	0	0	0	0	1	0	0	1
Thrombocytopenia	0	0	0	1	0	0	0	0	0	0	0	0	1
Microcephaly	0	0	0	0	0	1	0	0	0	0	0	0	1
Neonatal Jaundice	0	0	0	0	1	1	0	0	0	0	0	0	2
Respiratory Distress	0	0	0	0	0	0	0	1	0	0	0	0	1
Petechiae	0	0	0	0	1	0	0	0	0	1	0	0	2
Petechiae with Jaundice	0	0	0	0	0	0	1	0	0	0	0	0	1
**Symptomatic** (Total)	3	2	1	1	2	2	1	1	1	1	1	0	16
**Asymptomatic** (Total)	1	0	2	0	5	2	2	2	2	2	1	1	20
**Total** (Symptomatic+Asymptomatic)	4	2	3	1	7	4	3	3	3	3	2	1	36

The overall distribution of individual sub-genotypes in this study cohort was as follows: gH1 (7/11; 63.7%), gH2 (4/11; 36.3%), gB1 (4/10; 40%), gB2 (2/10; 20%), gB3 (3/10; 30%), gB4 (1/10; 10%); gN1, gN3-(a, b, c) and gN-4a (3/15; 20%), respectively; gN-4b (2/15; 13.3%) and gN-4c (1/15; 6.7%) as in Table 6.

The distribution of HCMV genotypes between symptomatic and asymptomatic children was analyzed to assess the possible association of genotypes with clinical signs and symptoms. Our findings revealed that compared to other, only gB1 genotype was significantly associated with hepatic damage when compared between symptomatic and asymptomatic neonates.

**Fig 2:**
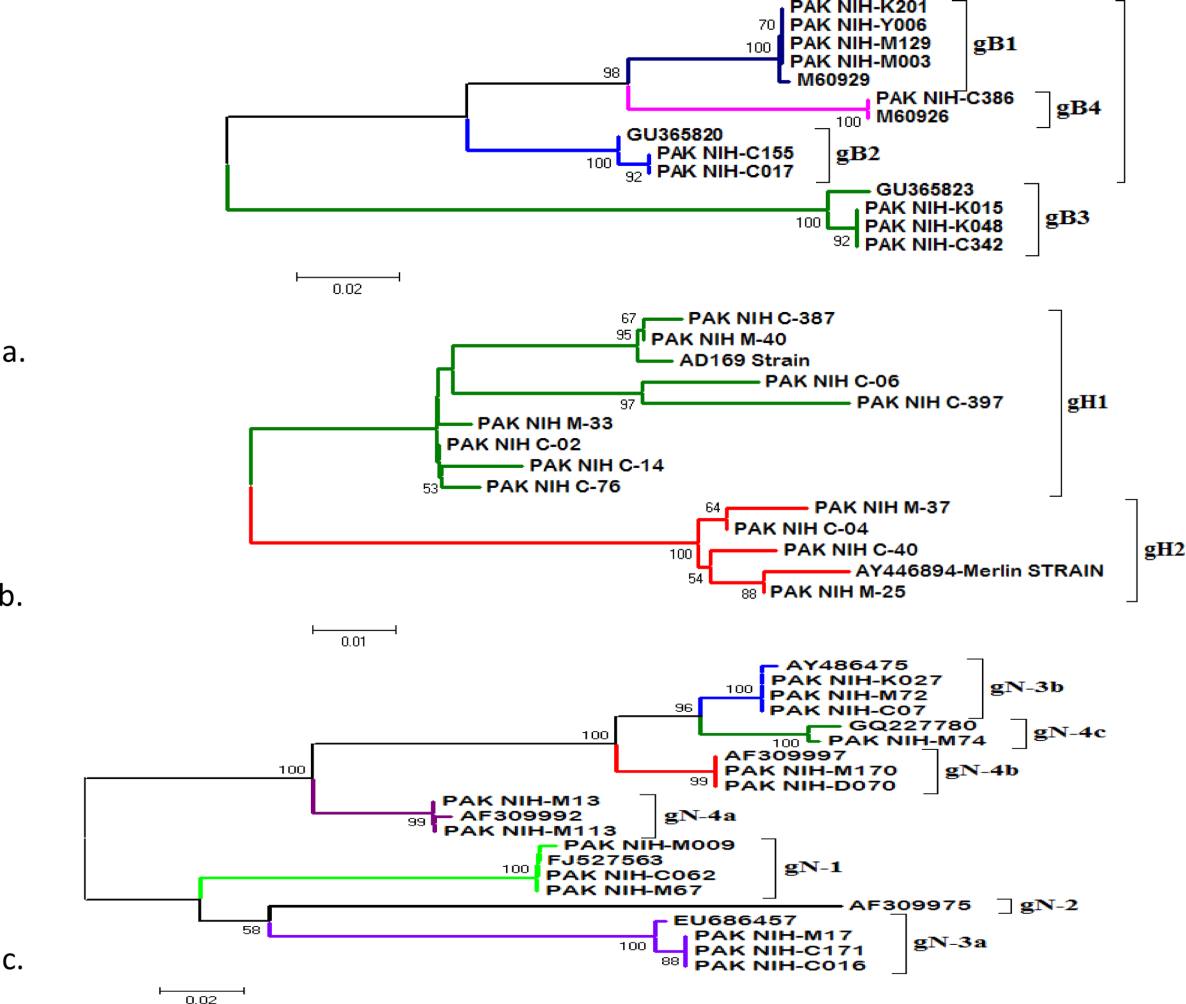
Phylogenetic analysis of gB, gN and gH genotypes of HCMV. The reference strains and isolates detected through BLAST are given for genetic comparison. The phylogenetic tree with 1000 bootstrap replicates was reconstructed using neighbor joining method and Kimura two-parameter distances model incorporated in MEGA v5.0. The number at the nodes indicates bootstrap values shown above 50. Fig 2A, 2B and 2C represent genetic relationships of gB, gH and gN genotypes of CMV respectively. The strains detected in this study are represented by sample identification with codes mentioned as PAK: Pakistan, NIH: National Institute of Health Islamabad.

The phylogenetic analysis showed that gB genotypes have 98.5 to 100% similarity with reference strains, gN genotypes showed 94.2 to 99.4% similarity and gH 95.3 to 99.7%, as shown in Fig 2.

## Discussion

HCMV is a frequent cause of congenital infection, the most common being sensorineural hearing loss, mental retardation, motor deficits, seizures, and chorioretinitis [19, 20]. The reason why only some children develop SNHL or other sequelae after congenital CMV infection is not well understood but could be related to both host and viral factors. At birth, it is essential to test for congenital CMV infection. Although congenital CMV infection occurs in 0.2 to 1% of live births worldwide, there has been no population-based HCMV clinical or epidemiological study in Pakistan. Majority of the Pakistani population lives below poverty level, and because health care facilities are not available, women are likely to be exposed to CMV infections. The main objectives of the present study were to investigate HCMV seroprevalence among expectant women, to explore the correlation of active infection to congenital infection in newborns and to determine the prevalent HCMV genotypes.

The seroprevalence of HCMV IgG in pregnant women in this study (97.55%) was comparable to a regional study carried out in the KPK province of Pakistan [21] as well as other countries such as Qatar [22], Egypt [23], Tunisia [24], Iraq [25], Iran [26], Brazil [27] and Turkey [28]. All these studies have reported higher seroprevalence rates than developed countries such as the United Kingdom [24] and Germany [29].

The testing of ELISA and PCR for CMV IgM antibodies and DNA revealed the positivity rates of 12.71% (n = 52/409) and 20% (n = 82/409) respectively. This may attribute to the selection of specific high risk group i.e. pregnant women. In addition, CMV positivity rate is inevitably high in the areas with high population density and low-resource settings like Pakistan [30]. It is likely that in developed countries, pregnant women are generally better informed of good hygienic practices such as hand washing and sanitation, thus accounting for a reduced risk of acquiring CMV infection. In the present study, only four (0.9%) of 409 women interviewed had heard about CMV infection previously. A lack of awareness about CMV infection among the public is a major barrier to controlling the disease. The high prevalence rate in our settings indicates the endemicity of infection and can be linked with socio-economic and climatic factors. In addition, the high frequency of women positive for HCMV DNA by PCR compared to ELISA reflects the higher sensitivity of nested PCR as reported previously [31].

In the current study, we observed that fever; jaundice and high parity were associated with active CMV infection. However, given the numerous causes of fever and jaundice, these represent non-specific indicators of maternal CMV infection. High parity increased the susceptibility to acquisition of maternal CMV infection, perhaps through direct contact with contagious secretions from their own children or poor hygiene practices [32, 33]. We did not demonstrate that active infection leads to congenital CMV infection, either symptomatic or asymptomatic, and did not clearly identify a relationship between the severity of disease and genotypes in neonates.

It is not entirely clear whether, over the course of the pregnancy, vertical transmission to the fetus after maternal viremia occurs either as a single event or through multiple transplacental transfer events. In this study, it was observed that CMV active infection during pregnancy led to intrauterine transmission and occurrence of congenital CMV infection as documented and reported by Boppana *et al* [34]. The majority of symptomatic cCMV infections were due to first trimester maternal infection, and the rate of sequelae in infected offspring is lower with maternal infection later in pregnancy. Daiminger *et al* [35] reported an association between earlier gestation maternal or fetal infection and poor outcomes for the fetus. Nevertheless, second and even third trimester maternal infections are also capable of causing hearing defects. However, the relationship between HCMV genotypes and the consequences of infection in terms of pathogenesis and long term outcomes in children with congenital HCMV infection are not yet clearly understood.

Classification of genotypes was performed by sequence comparison of glycoprotein gene from Pakistani strains using online BLAST (Basic Local Alignment Search Tool) analysis. Genotype gB1 was recently found to be associated with severe manifestations of CMV disease in infants with congenital CMV infection [36]. Other studies suggest that the gB, gN and gH genotypes of CMV may result in neurological dysfunction or hearing loss in congenitally infected infants [37, 38]. Our findings also support these studies and further demonstrate that gN and gH genotypes may be associated with congenital infection [39, 40]. Pignatelli *et al* [17] described the distribution of gN (UL73) variants gN-1, gN-3 and gN-4 and their subtypes in four geographical regions; Europe, China, Australia and North America with different frequencies of gN-2 (rarest genotype), which was found in North America and Europe but was not identified in Asian countries and Australia. Likewise, we did not find any gN-2 genotype in study confirming the previous findings from this region.

Urine and saliva specimens have been suggested for the detection of congenital HCMV infection as sample collection from neonates is simpler as compared to blood; higher volumes can be easily obtained and the procedure can be repeated easily if needed as parent compliance is higher. Out of the total urine samples collected from 18 neonates, 5 were negative although saliva from all of these neonates was positive. Thus our study confirms previous findings that testing saliva is more sensitive than urine for CMV testing [41].

The prevalence of different genotypes in symptomatic and asymptomatic infants was not significantly different, and none of the genotypes were associated with virulence. Although no significant genotype specific-association with specific clinical features could be established, all the infants with hepatosplenomegaly carried the gB1 genotype, which has been reported globally by other groups [36, 42]. However, our study contradicts other publications which reported that only gB genotypes were associated with virulence [36, 42, 43]. This may be due to differences in geographical location, population selection or other factors such as culture-propagated viral isolates which could select for a single virus strain and thus decrease the chances of identifying multiple viral strains or genotypes [17, 44]. Many other studies have also described HCMV infection with multiple viral strains in different populations, including children attending day care centers, HIV-infected individuals, allograft recipients, and infants with congenital HCMV infection [44–47]. Together, these findings suggest that infections with multiple CMV strains occur frequently.

Our study thus represents the first report of CMV infection and its circulating genotypes in Pakistani population focusing the primary risk group i.e. pregnant women. This may underestimate the true burden of HCMV in indigenous Pakistani population. Likewise, we were able to target only two major hospitals in Islamabad that may not represent the true disease burden and outcomes in terms of congenital infections for the entire Pakistani population. Due to limited number of samples within a given population, the comprehensive epidemiology of HCMV genotypes and their clinical impact cannot be truly established. Thus the study findings emphasize continuing large scale epidemiological surveys in the other areas of the country. Public awareness programs must been introduced and scaled-up targeting the population at risk and those in the regions with high population density and low hygiene and socio-economic status.

## Conclusion

Active CMV infection during pregnancy was a major cause of cCMV infection, and distribution of gB, gN and gH genotypes were similar in symptomatic and asymptomatic neonates. Currently, no therapeutics or vaccines are available for CMV; therefore, minimizing the number of cases of CMV by informing women about their CMV serostatus before and during pregnancy and educating them about preventive hygienic measures to avoid exposure to CMV is crucial. It is difficult to estimate the true epidemiological profile of CMV infections and prevalent genotypes in our population due to absence of routine screening and surveillance studies. Indeed, there is a dire need for early diagnosis of CMV to reduce congenital CMV infections and to determine the true burden of the disease. The availability of current information on CMV prevalence and implementation of disease control measures will ultimately enable us limit exposure to the virus and reduce childhood mortality as envisaged for the Millennium Development Goals.
